# A review of non-invasive sensors and artificial intelligence models for diabetic foot monitoring

**DOI:** 10.3389/fphys.2022.924546

**Published:** 2022-10-21

**Authors:** Maria Kaselimi, Eftychios Protopapadakis, Anastasios Doulamis, Nikolaos Doulamis

**Affiliations:** National Technical University of Athens, School of Rural, Surveying and Geoinformatics Engineering, Athens, Greece

**Keywords:** diabetic foot, artificial intelligence, review, sensors, hyperspectral imaging

## Abstract

Diabetic foot complications have multiple adverse effects in a person’s quality of life. Yet, efficient monitoring schemes can mitigate or postpone any disorders, mainly by early detecting regions of interest. Nowadays, optical sensors and artificial intelligence (AI) tools can contribute efficiently to such monitoring processes. In this work, we provide information on the adopted imaging schemes and related optical sensors on this topic. The analysis considers both the physiology of the patients and the characteristics of the sensors. Currently, there are multiple approaches considering both visible and infrared bands (multiple ranges), most of them coupled with various AI tools. The source of the data (sensor type) can support different monitoring strategies and imposes restrictions on the AI tools that should be used with. This review provides a comprehensive literature review of AI-assisted DFU monitoring methods. The paper presents the outcomes of a large number of recently published scholarly articles. Furthermore, the paper discusses the highlights of these methods and the challenges for transferring these methods into a practical and trustworthy framework for sufficient remote management of the patients.

## 1 Introduction

Nowadays, millions of people worldwide are living with diabetes. Mistreated diabetes may lead to adverse situations, including the development of diabetic foot ulcers (DFU) or appearance of regions susceptible to infection (Jaly et al., 2020). Infections can range from mild (e.g., cellulitis) to severe ones, such as those affecting the bones (e.g., osteomyelitis). A severe infection can lead to a life-threatening emergency situation (e.g., sepsis), that requires treatment with antibiotics, administered intravenously, and surgical intervention for drainage, debridement, or amputation (Lim et al., 2017). Nevertheless, these cases could be prevented through regular assessment (Cousart and Handley, 2017).

On the one hand, DFU is treated by specialized medical experts. On the other hand, patient’s self-care, outside the clinic, is of utmost significance (Bus and van Netten, 2016). In that context, patient empowerment towards self-monitoring is considered of high-importance. Self-management and monitoring can contribute in preventing an initial ulcer appearance, detecting susceptible signs in a foot region, monitoring existing ulcers to prevent further complications and possible recurrent ulcerations (Armstrong et al., 2017).

There are multiple signs/symptoms related to diabetic foot ulcer. These signs involve skin color change (redness), temperature change, damage to the skin due to abnormal foot plantar pressure, change in pain level or appearance of a new pain, swelling, or odor (Schaper et al., 2016). Most of these indication signs can be captured and, thus, monitored, using various optical and/or laser sensors. Nowadays, red-green-blue (RGB) and thermal sensors have relatively low acquisition costs, are not bulky, and can be integrated to portable devices. Sensory inputs, coupled with deep learning (DL) models, can provide robust mechanisms for preventing undesirable or emergency situations (Tulloch et al. (2020)).

DFU monitoring and prevention is an active research field and there are many important publications in the area of study. Machine learning models including both, shallow learning, and DL approaches have been widely used to support DFU monitoring techniques. (Goyal et al., 2020). Yet, to the best of our knowledge, a sensor-driven survey, spanning multiple monitoring capable ranges, is missing. In this paper, we investigate how smart, low-cost devices (Najafi et al., 2020) embedded with AI tools, could contribute to self-prevention and monitoring of the DFU. This survey focuses on two research topics: a) DFU pathology monitoring, through optical sensors and b) analysis/interpretation of the sensor-oriented data, using machine learning/AI tools.

The remaining of the paper is organized as follows: Section 2 describes the basic monitoring areas, regarding the DFU pathology and clinical features related to DFU detection and monitoring. Sections 3 summarizes the noninvasive sensors that are utilized in DFU monitoring, whereas Section 4 is a brief overview of the machine learning techniques that are applied in DFU monitoring. Section 5 summarizes and analyzes the sensors along with the machine learning techniques that are utilized focusing on Near-Infrared (NIR), Mid-Infrared (MIR) and Long-Infrared (LIR) clinical studies. Section 6 concludes this work and highlights the challenges towards a practical and trustworthy framework for self-monitoring, AI-assisted DFU monitoring approaches.

## 2 DFU monitoring fundamentals

### 2.1 DFU development risk factors

DFU is characterized by a complex multifactorial pathogenesis. In the following paragraphs, the most prevalent risk factors and parameters for foot ulcer recurrence (Armstrong et al., 2017) are briefly described, since the medical history of the patient is related to DFU development and progression. The risk factors include: a) the duration of diabetes, b) the history of vascular intervention, c) the amputation, and d) the existence of callus. In the same study, factors such as age, gender, body mass index, smoking, nephropathy, tinea pedis, and hyperkeratosis were not related to DFU.

At first, the duration of diabetes and the blood glucose levels are crucial factors and, thus, consist a key indicator for diabetic foot monitoring (Cheng et al., 2021). Secondly, loss of sense in the foot, due to diabetic neuropathy, in combination with foot deformities, poorly fitting footwear, and excessive pressure, can result in callosities formation that may lead to ulcers development. Thirdly, the peripheral arterial disease (PAD) can cause a decrease in blood circulation, which may cause ulceration, or delay foot healing by reducing the oxygen delivery to peripheral tissues. PAD maym, also, cause a reduce in temperature on plantar foot. Lastly, oxygen concentration can be indirectly calculated; given that hemoglobin-associated oxygen accounts for roughly 97% of total oxygen being transported, the dynamic relationship between oxygen and hemoglobin levels can be assessed for primary determination of oxygen transport.

As such, clinical studies suggest that patients, who have developed or are at risk of developing a diabetic foot ulcer, should periodically monitor and assess the following four factors: a) blood glucose levels (Kateel et al., 2018), b) foot deformity/foot pressure (Yazdanpanah et al., 2018), c) foot temperature (Mejaiti et al., 2018) and d) hemoglobin concentration (Salman et al., 2017). Moreover, the duration of diabetes, the medication history of the patient, and the history of vascular interventions (if any) are also important factors for foot ulcer recurrence.

### 2.2 DFU clinical features

A diabetic foot ulcer is an open wound with a circular appearance, located on the bottom of the foot and often preceded by a haemorrhagic subepidermal blister. Tissue around the ulcer may become black, and in some cases may gangrene will developed. Foot ulcers are usually painless, leading to delay visits to health professionals. Pedal pulses may be absent and reduced sensation can be demonstrated.

## 3 Non-invasive sensor technologies for DFU monitoring

The widespread acceptance of non-invasive sensor technologies is essential for the following reasons: a) to monitor major risk factors associated with diabetic foot ulcer, b) to empower patients in self-care, and c) to effectively deliver the remote monitoring and multi-disciplinary prevention needed for those at-risk people and d) to address the health care access disadvantage that people living in remote areas (Najafi and Mishra (2021)). Harnessing sensor technologies to remotely manage diabetic foot is of major importance. In this section we summarize both the sensor technologies and spectrum’s rage that had been used to this day, for monitoring purposes.

### 3.1 Techniques in the visible spectrum

Visible spectroscopy (VIS) is defined by the luminous efficiency functions ranging between wavelengths of *λ* = 380 *nm* and *λ* = 780 *nm* (Salman et al., 2017). These sensors measure the surface wound size, or identify the wound boundary/area, since they are visible effects under the light. Moreover, RGB sensors distinguish multiple types of skin deformities (van Netten et al., 2017). Therefore, digital images are utilized for monitoring the wounds’ characteristics.

**FIGURE 1:**
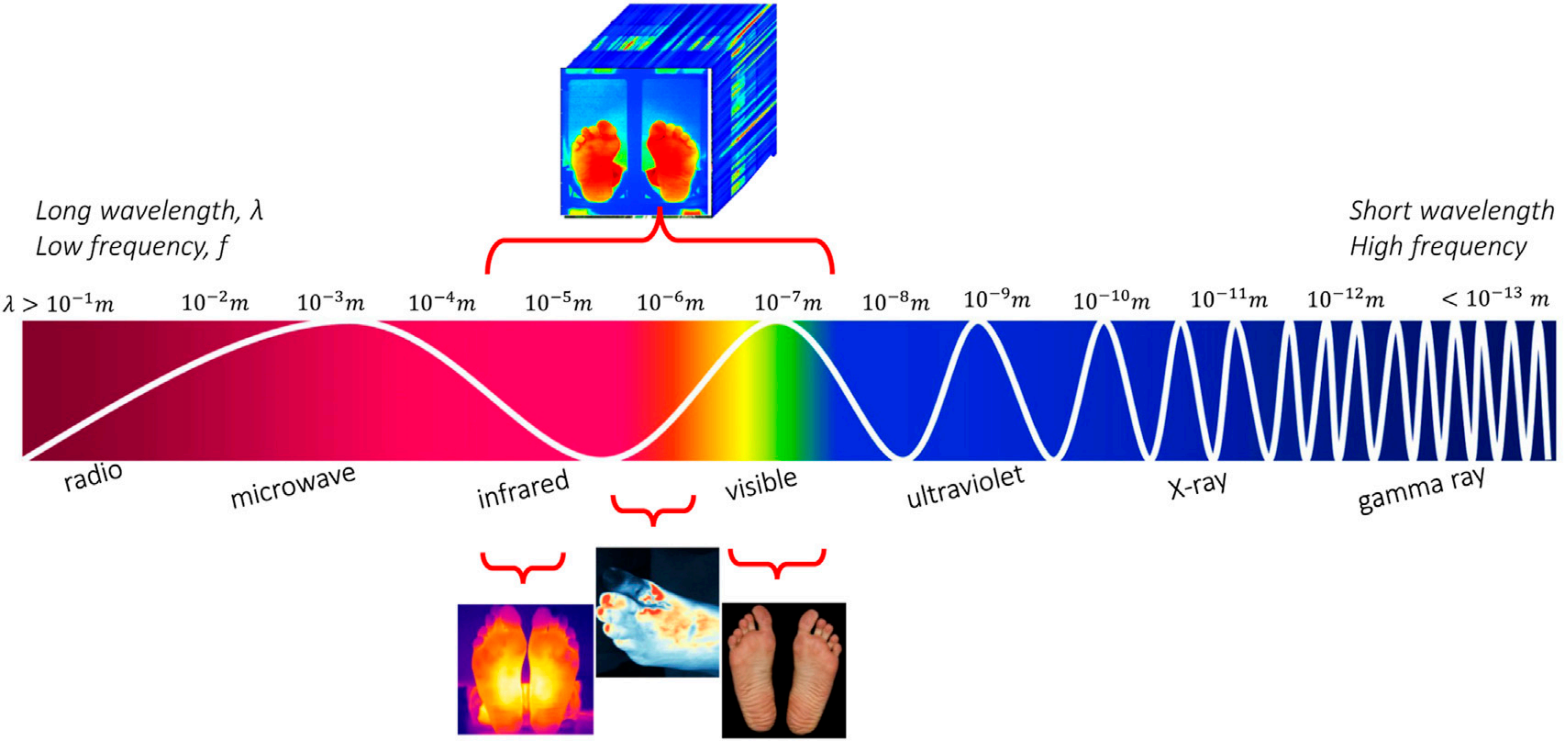
The electromagnetic spectrum along with the imagery techniques for the diabetic foot monitoring.

RGB images are usually identified in computer applications, hand-held devices and mobile applications for wound assessment and monitoring. Monitoring devices equipped with RGB (red-green-blue) sensors is a low cost solution, which can be applied easily for self-monitoring or in primary prevention. However, they do not yield oxygenation or birefringence-related characteristics compared to other approaches. VIS cannot support temperature, glucose, or hemoglobin concentration monitoring. Nevertheless, if we move beyond the visible spectrum, near-/mid-/far-infrared wavelengths can support clinical diagnostics. Figure 1 illustrates the ranges of sensors under consideration in this study. Spectral information other than optical can also be referred to as hyperspectral.

### 3.2 Techniques in the near-infrared spectrum

NIR spectroscopy provides a viable approach for monitoring saturation levels under the skin. Tissues are relatively transparent to near-infrared light and the absorption of light dependents on the oxygenation status of the tissue (Nouvong et al., 2009). Furthermore, the absorption, scattering, and reflection properties of the propagation of light in a tissue are affected by the wavelength. Yet, the difference between oxygenated and deoxygenated blood can be easily identified in the NIR spectrum. In particular, NIR spectroscopy (700*nm* − 1300 *nm*) captures hemoglobin saturation (*HbO*_2_, *Hb*) and peripheral/tissue oxy saturation (*StO*_2_, *SpO*_2_), or deoxy levels, which are important indicators for DFU early detection (Xie et al., 2021).

### 3.3 Thermal infrared imaging technologies

Infrared (IR) imaging techniques are utilized for tissue assessment. IR detectors produce a heat map of the foot surface and are utilized for the assessment of the temperature variations in the plantar region. Thermal imaging has been previously used for the diagnosis of many medical conditions, including skin/breast cancer, arthritis, allergy, burns, among others (Gurjarpadhye et al., 2015).

### 3.4 Hyperspectral imaging technologies

Hyperspectral imaging (HSI) provides information about the chemical properties of materials and their spatial distribution (Khan et al., 2018). HSI covers a continuous portion of the light spectrum, including infrared, near-, mid- and far-infrared (FIR) spectral ranges. Thus, HSI captures the subtle spectral differences under different pathological conditions (Fei, 2020), contrary to multispectral imaging, which supports a limited number of discrete spectral bands. HSI data structures involve multiple 2*D* images (spatial information) at discrete wavelengths (spectral information) to produce a hypercube (*x*, *y*, *λ*); each pixel in the image is described by a diffuse reflectance spectrum. However, low-cost smartphone-based hyperspectral imaging systems are not mature yet, even though there is a rapid progress in this field Stuart et al. (2021). Thus, hyperspectral imagery data sources provide rich information for different ulcer characteristics, however the equipment that is needed is expensive.

### 3.5 Comparison of the different optical imaging techniques

Considering the major factors to monitor and the capabilities of the previously described sensors, we could advocate favorably that diabetic feet monitoring using non-invasive sensors is a feasible solution. Yet, successful monitoring requires the combination of different types of sensors, covering multiple ranges, including VIS, near-, mid-, and far-infrared. Table 1 summarizes the range of wavelengths for the investigated sensors.

**TABLE 1:** Sensing techniques for DFU monitoring.

Non-invasive sensor technique	Measurements/Features of interest
RGB imaging	clinical features of the diabetic foot complications (epidermal thickness, melanin concentration, ruddiness)
Hyperspectral imaging	oxygen saturation of foot tissues
Thermal imaging	temperature variations
Techniques in the near-infrared spectrum	hemoglobin saturation (HbO2, Hb) and peripheral/tissue oxy saturation (StO2, SpO2), or deoxy levels

VIS (400–700 *nm*) captures different skin color and texture properties. NIR spectroscopy (700*nm* − 1300 *nm*) can capture hemoglobin saturation (HbO2, Hb) and peripheral/tissue oxy saturation (StO2, SpO2), or deoxy levels, which are important indicators for DFU early detection (Xie et al., 2021). Thermal Infrared spectroscopy can detect hyperthermia (or hypothermia), among ROIs of a foot (or comparatively between right/left foot), that consist major factors for microcirculation and edema assessment in DFU (Rubins et al., 2019). Finally, the Mid-IR absorbance (5.7 *μm* − 9.3 *μm*) spectrum contains rich information about the proteomics, lipidomics and metabolomics (e.g., glucose) (Kottmann et al., 2012).

## 4 Machine learning techniques for DFU monitoring

### 4.1 Overview of the machine learning techniques for DFU

#### 4.1.1 Supervised learning techniques

Machine learning and signal processing methods can contribute significantly in early diagnosis, predictive modelling, analytics, and characterization of the diabetic foot (Tulloch et al., 2020; Yap et al., 2021). ML approaches focusing on DFU detection have received renewed attention, mostly thanks to the increased number of datasets with RGB images (Yap et al., 2022). To this day, various models have been developed using supervised learning methods to detect/localize abnormalities on medical images related to diabetic foot (Zhang et al., 2022). These tasks may involve: (i) classification (Goyal et al., 2020; Xu et al., 2021), (ii) object detection (Cassidy et al., 2021), and (iii) semantic segmentation (Rania et al., 2020; Kendrick et al., 2022). Depending on the scenario, different types of training sets are used, and a minimum but sufficient amount of data is required.

Nowadays, research depends, mainly, on deep learning approaches for solving complex problems. The main advantage lies in the capability to discover hidden patterns in the data or establish a better understanding of intricate relationships among many interdependent variables. Given the sensors availability, and the spectral bands range per sensor, we have large amounts of data appropriate for training purposes. Consequently, deep learning approaches occurred naturally as an application scenario in DFU monitoring, based on optical information (Voulodimos et al., 2018).

In medical applications, the availability of labeled training data is extremely limited owing to the nature of the data containing protected health information. However, supervised machine learning requires a great amount of labeled data to train a model, which is at the origin of the main bottleneck in model development. In literature, the studies address the lack of labeled data by proposing alternative methods to overcome this problem (Alzubaidi et al., 2021). As shown in sub-sections that follow, unsupervised or semi-supervised schemes are efficient for these scenarios.

### 4.1.2 Unsupervised learning techniques

Unsupervised learning based models have been used to surpass the problem of labelled data, since these algorithms can infer adequate feature representations of input values, without using labelled data. Typically, in healthcare related studies unsupervised learning manifests as clustering techniques, which detect hidden patterns or groupings in data Tulloch et al. (2020). Extracting meaningful features from the original raw values is useful for reducing the dimension of feature space and achieving better clustering performance Ji et al. (2020).

### 4.1.3 Semi-supervised learning techniques

Other approaches may involve tensor-based Makantasis et al. (2021) and semi-supervised learning Protopapadakis et al. (2021). Both techniques exploit feature space projections, which allow for better handling of sets of high-order data and/or limited training sets. The former case, i.e., tensor-based learning, imposes canonical/Polyadic (CP) decomposition of rank-R on its weights, leading to a significant reduction of the number of hyperparameters during training. The later case, couples labeled and unlabeled data to form additional regularization terms, resulting in higher performance models.

### 4.2 Identified sensor/AI tools synergies

Diabetic foot can cause severe morbidity in diabetic patients with a subsequent increased cost for the health care system. Prevention and early diagnosis are the keys to decrease the prevalence of diabetic foot. Due to Covid-19 pandemic evolution, public health guidelines should be reconfigured to support and manage diabetic foot patients including remote consultations. Self-foot assessment is a critical tool for the highly beneficial early detection of ulcers. Development and proper usage of an objective tool that can assess the foot of a diabetic patient are key interventions towards prediction and early diagnosis of diabetic foot with significant benefits for the patient and the health care system. Various sensory systems can be used towards this direction. Table 2 highlights the most important conclude remarks per sensory system.

**TABLE 2:** Synergies between machine learning tools and non-invasive methods for DFU monitoring.

Noninvasive sensor technique	Measurements	Algorithms	Sensors/Cameras	Criteria
RGB imaging	color, size, shape, texture in DFU ulcers	Supervised object detection (region of the ulcer) or binary classification algorithms (health or ulcer)	Kodak DX4530, Nikon D3300 and Nikon COOLPIX P100	Sensitivity, specificity, precision, accuracy, F-Measure, AUC score for accurate detection or classification
Infrared/Thermal imaging	Temperature	Classification or clustering techniques, image processing and edge detection algorithms	Pixels (320*H* × 240*V*), measuring range (starts from −20 °*C* up to +150 °*C*), spectral range (8.0 *μm*–14.0 *μm*), field of view (1.2 *μRad*), resolution (>16bit)	Temperature differences in foot: ulcerous *IF* > 2.2 °*C* and noninfected and nonischemic foot ulcer IF <2.2°C
HSI	Oxygen saturation of foot tissues	Unsupervised techniques (PCA) for dimensionality reduction and ML classification techniques (e.g., SVM, ANN, CNN, *etc.*)	Spectral range: 400–720 *nm* Detector: CCD Dispersive device:LCTF Acquisition Mode: Staring Measurement Mode: Reflectance	Differences (D) in oxyhemoglobin [D (OXY)] >18 and deoxyhemoglobin [D (DEOXY)] >5.8 between the target and the neighbor regions are significant
Mid-IR spectroscopy	Glucose concentration	Signal processing and statistical methods, no ML methods yet	Quantum Cascade Laser (EC-QCL). QCL MIR glucose absorption	glucose concentration more than 100 *mg*/*dl*

Abbreviations are listed in Appendix A.

## 5 Grouping of the machine learning approaches in DFU monitoring depending on the range of the electromagnetic spectrum

Non-invasive sensors have contributed in a great extend for diabetic foot monitoring. In addition, the recently released datasets captured from these sensors provide a breeding ground for the application of machine learning algorithms. In this section, we group the literature works and the proposed approaches, based on the type of sensor that is utilised during capturing and the electromagnetic spectrum that is covered. In particular, we divide the literature work in four main categories that is works dealing with: i) data captured from the visible spectrum, ii) hyper-spectral imaging data, iii) thermal imaging data, and iv) data from the middle-IR spectrum.

In every single subsection, the respective research works are structured in a table format. The columns of each table are: i) an identifier of the presented work, ii) information about the equipment that is utilized from data capturing as well as information about the environmental conditions during the data capturing process, iii) the number of the participants in the clinical study, their inclusion/exclusion criteria if available and the participants number in total, in the forth column we have iv) the adopted modelling approach/pipeline and finally, we present v) the experimental results, the utilized metrics and the final findings of the study.

### 5.1 AI techniques and imagery data of the visible spectrum

In this subsection, we summarize the literature works that process imagery data of the visible spectrum (RGB). The research interest in DFU is growing, as the number of reported cases of diabetes is also grown at a worldwide level. Early attempts in training machine learning models in this domain have shown promising results. Wang et al. (2019) use four different support vector machines (SVMs) to determine wound area in images. Yap et al. assessed the reliability of an application to standardize the image capture of DFUs Yap et al. (2018). Nanda et al. (2022) evaluate the performance of different shallow learning techniques, including Random Forests (RF), SVM, Naive Bayes (NB), K-nearest neighbour (KNN) methods, for the identification of the risk factors associated with development of diabetic foot ulcer. Recently, the release of the Diabetic Foot Ulcers Grand Challenge (DFUC 2020) dataset consisting of labelled images has attracted the interest for applying machine learning algorithms in diabetic foot monitoring applications. The dataset consists of 2,496 ulcers in the training set and 2,097 ulcers in the testing set.

With the entrance of new labelled datasets, the implementation of deep learning approaches is feasible. As regards the classification, in the work of Alatrany et al. (2022), they used a deep learning autoencoder as a feature extractor and subsequently trained multiple ML models to accurately classify healthy and DFU skin regions. Feature fusion techniques that combine low- and high-level information could be combined as proposed in Das et al. (2022), to improve the capabilities in identification of DFU normal and abnormal classes. As regards semantic segmentation tasks, Cui et al. compare SVM algorithms among with two convolutional neural networks (CNN)-based network architectures (U-Net and a patch-based CNN), showing that the U-Net approach yields the best performance compared to others Cui et al. (2019). Another work that supports these findings is the work of Ohura et al. (2019), who compared four different CNN architectures and identified U-Net as the best performing method, with sensitivity of 85.8% and specificity of 98.8% on the DFU dataset. Recently, the modern implementations in DFU detection methods include the popular deep learning models for object detection, such as Faster R–CNN, YOLOv3, YOLOv5, and EfficientDet (Yap et al., 2021). Also, ensemble method are proposed in the literature as well as networks consisting of cascade attention networks ((Cai and Vasconcelos, 2019)).

Table 3 summaries the literature works where RGB imaging data used for diabetic foot applications, along with the analysis procedure followed and the findings of each study. Both shallow and deep learning methods are considered.

**TABLE 3:** Summary of the literature for diabetic foot monitoring using RGB imagery data.

Study	Basic Equipment and Environmental Conditions	Participants (number and characteristics) or dataset description	Algorithms and methods	Experimental Results and performance evaluation
Cui etal.,2019	high resolution images of the wound provided by New York University (NYU)	445 RGB images	CNN segmentation (U-Net)	The proposed method achieves precision = 0.768, sensitivity = 0.937, specificity = 0.960
Ohura etal.,2019	-	-	various segmentation models (such as, SegNet, LinkNet, UNet and UNet–VGG16)	U–Net achieves accuracy = 0.997, specificity (0.943) and sensitivity (0.993)
Goyal etal.,2018)	3 RGB cameras (Kodak DX4530, Nikon D3300 and Nikon COOLPIX P100)	1775 foot images with DFU collected from the Lancashire Teaching Hospitals	Faster-RCNN and R-FCN deep learning methods. DFU regions detection	Mean average precision (mAP) considering a correct detection of foot ulcer. *mAP* = 90.1%
Cassidy etal.,2021	3 RGB cameras (Kodak DX4530, Nikon D3300 and Nikon COOLPIX P100)	Diabetic foot images from the Lancashire Teaching Hospitals including in total 1775 diabetic foot images	Faster-RCNN YOLOv5 EfficientNet	Mean average precision (mAP) considering a correct detection of foot ulcer. *mAP* = 0.66% [Faster R-CNN]
Davradou etal.,2022	-	DFUC 2020 dataset 4,000 images, with 2000 used for train	super resolution and denoising using DL models	RMSE, PSNR and SSIM metrics. *SSIM* = 0.93% [ISR model]
Tzortzis etal.,2022	-	DFUC 2020 dataset 2000 images, with 1,000 used for train	Spectral, kmeans and Meanshift region clustering on DL features	Cluster based metrics and IoU. *IoU* = 0.7%

### 5.2 AI techniques and hyperspectral imagery data

Near-infrared radiation can be transmitted through the body, since it is not absorbed by water or hemoglobin Pasquini (2003). The near infrared spectra consist of vibrational overtones and combination absorption features with spectral signatures that allow identification and mapping of different materials. Oxygenation/deoxygenation of hemoglobin, oxidized cytochrome c oxidase, and oxygenated/deoxygenated myoglobin have a unique absorption spectrum in the NIR region Ciurczak and Igne (2014). The diagnostic potential of NIR spectroscopy has been previously used to study a range of diverse conditions, including diabetes mellitus chronic complications Aitchison et al. (2018), atherosclerotic occlusive disease Saito et al. (2018), Alzheimer’s disease Khagi et al. (2018), *etc.*

Triggered by the different absorption spectra of oxygen and deoxyhemoglobin, in biomedical HSI, most of the researchers have utilized reflectance spectra to estimate oxygen saturation (SpO2) values from peripheral tissue (Yang et al., 2018). Hyperspectral tissue oxygenation measurements can readily indicate changes in tissue surrounding the ulcer, when comparing ulcers that heal and ulcers that do not heal.

Hyperspectral data have various spectral bands. However, due to the curse of dimensionality, the existence of multiple spectral bands may decrease the performance of the classification algorithms (Zhang et al., 2020). Principal component analysis (PCA) is a common technique for dimensionality reduction in medical hyperspectral datasets.

Then, ML algorithms automatically classify ulcers as healing or non-healing. Techniques, such as SVMs and neural networks (NN) or even CNNs, are widely used for medical hyperspectral image classification. HSI technique was used for healing prediction in routine practice. The data were analyzed to detect differences between patients with DFUs that healed and those with DFUs that did not heal. Table 4 summarizes the research on diabetic foot monitoring in the near infrared spectrum.

**TABLE 4:** Summary of the literature for diabetic foot monitoring using hyperspectral imagery data.

Study	Basic Equipment and Environmental Conditions	Participants (number and characteristics) or dataset description	Algorithms and methods	Experimental Results and performance evaluation
Yudovsky etal.(2010)	Characteristics: (i) seven broadband visible LEDs (ii) a spectral separator (LCTF-10–20) tunable over the range of 400–720 nm (iii) a CCD and (iv) a 25 − *mm* focal-length imaging lens	66 volunteers at the Olive View Medical Center (Olive View-UCLA IRB No. 05H-609300)	Binary classification between healing and non-healing ulcers. Two classes are created: ulcers that healed within 24 weeks and (ii) ulcers that did not heal within 24 weeks	Tissues at risk of ulceration with 0.95 sensitivity for images were taken 58 days before tissue damage is apparent to the naked eye. The maximum differences in oxyhemoglobin |*MD* (*OXY*)| > 18 and in deoxyhemoglobin |*MD* (*DEOXY*)| > 5.8 are statistically significant
Khaodhiar etal.,2007	HyperMed CombiVu-R System uses a spectral separator. The spatial resolution of the HT images was 60*m*	10 type 1 diabetic patients (21-foot ulcer sites) and 13 type 1 diabetic patients without ulcers and a control nondiabetic group with 14 participants. Visits 4 times over a 6-month period	Oxyhemoglobin (HT-oxy) and deoxyhemoglobin (HT-deoxy) measurements at or near the ulcer area and on the upper and lower extremity distant from the ulcer. An HT healing index for each site was calculated	HT oxygenation measurements, comparing healed and non-healed ulcers (*p* < 0.001). Changes in HT-oxy for all the three risk groups in the metatarsal area of the foot (*p* < 0.05) and the palm (*p* < 0.01)
Yang etal. (2018)	HSI setup with: Illumination of the foot was 16 × 1 W white LEDs with 8 units. Push-broom type. A Peltier cooled CCD coupled to an imaging spectrograph. 3D data cube contained 2D spatial images (120*x*170 pixels) over a 430–750 wavelength range	43 volunteers in total. 12 women and 31 men; mean age was 62.7 years 6 out of 43 had type 1 diabetes and 37 had type 2 diabetes; 9 were smokers and 39 patients have neuropathy	Hyperspectral images of 43 patients were analysed using the *SpO*_2_ data processing and PCA techniques	PCA (sensitivity = 87.5% and specificity = 88.2%) outperformed SpO2. ROC analysis revealed an area under the curve of 0.88 for PCA compared with 0.66 using *SpO*_2_
Greenman etal. (2005)	HSI uses a spectral separator. HyperCal-1 calibrator. The spatial resolution of the MHSI images was 60 *μm*	108 patients (21 control non-diabetic individuals, 36 diabetic patients who did not have neuropathy and 51 patients with both diabetes and neuropathy)	Haemoglobin saturation (*S*(*HSI*)*O*_2_ through medical HSI imaging; % of oxyhaemoglobin in total haemoglobin) in the forearm and foot	In the foot at resting *S*(*HSI*)*O*_2_ was higher in the control (38 [*std* = 22]) and non-neuropathic groups (37 [*std* = 12]) than in the neuropathic group (30 [*std* = 12], *p* = 0.027)
Jeffcoate etal.(2015)	-	43 patients (37 Type 2 and 6 Type 1 diabetes) with foot ulcers were included in the clinical study	Tissue scattering of light and validation using blood samples of varying oxygen saturation and blood gas analysis	Strong correlation between the results of HSI and blood gas analysis (*r* = 0.994). Positive correlation between oxygenation and time to healing was observed (*p* = 0.03)
Nouvong etal.(2009)	HSI system uses wavelengths between 500 and 660 *nm* to include oxy and deoxy absorption peaks	54 patients (with type 1 or type 2 diabetes) with 73 ulcers; at 24 weeks, 54 ulcers healed while 19 ulcers did not heal	Linear discriminant analysis was used to develop the threshold for separating the healed and non-healed DFU groups	Healing index to predict healing with 0.8 sensitivity, 0.74 specificity and 0.9 positive predictive value. Correlation between cutaneous tissue oxygenation and wound healing in diabetic patients

### 5.3 Mid-infrared sensing for DFU

Mid-IR photoacoustic (PA) spectroscopy techniques utilize wavelengths of light in a range that allows monitoring glucose concentration levels in epidermal skin. However, MIR spectroscopy does not penetrate in deep skin layers and it is usually observed signal deterioration due to the strong absorption of water, which is in abundance in human body. To overcome this limitation, Kottman et al. Kottmann et al. (2012) proposed a hybrid setup that consists of a photoacoustic device and a tunable quantum cascade laser (QCL) to track glucose in deep epidermal layers. This dual-wavelength approach yields a considerably improved stability and lower uncertainty compared to the traditional MIR spectroscopic techniques (Table 5).

**TABLE 5:** Summary of the literature for diabetic foot monitoring using mid-infrared spectroscopy.

Study	Basic Equipment and Environmental Conditions	Participants (number and characteristics) or dataset description	Algorithms and methods	Experimental Results and performance evaluation
Kottmann etal.,2012	Tunable Quantum Cascade Laser (EC-QCL), continuous-wave laser light. QCL chip covers a range of glucose absorption in the Mid-IR. The maximal average laser power 20–130 *mW*	Patients/children from the University Childrens Hospital of Zurich	Glucose detection in epidermal skin samples. Decision support methods utilized	The photoacoustic signal linearly depends on the glucose concentration within the large concentration range of 0 *to* 10 *g*/*dl* the dual-wavelength approach yields an uncertainty of ±30 *mg*/*dl* of the blood glucose concentration level with a confidence level of 90%

### 5.4 Infrared thermal sensing for DFU

Long Wavelength Infrared cameras can be utilized for temperature inspection applications, due to their capability in detecting distinct temperature differences. Infrared thermography (IRT) is a fast, passive, contactless and non-invasive technique for temperature monitoring, for various parts of the human body (Vardasca et al., 2019a). Body’s temperature distribution is an important indicator for various disease patterns, and thermography stands as an adequate and flexible procedure, which is easy to use and has a low-cost (Sarawade and Charniya, 2018).

A thermal camera consists of five components: the optic system, the detector, the amplifier, the signal processing component, and the display (Sarawade and Charniya, 2018). Medical Infrared Thermography output is a two-dimensional digital image that provides data about the physiology of tissues (Hillen et al., 2020). Currently, multiple studies focus on the diagnosis of diabetic foot diseases using skin temperature variation (van Netten et al., 2013). A commonly adopted approach involves the division of the images into Regions of Interest (ROIs) and, then, the comparison of the relative difference in temperature, between the right and the left foot respectively.

Given the thermal imaging of the two feet, the mean temperature difference (Δ*T*) between each ROI of the right foot and the respective ROI of the left foot is calculated. Having computed the mean absolute temperature difference between corresponding points in both feet, various machine learning techniques are proposed for automatic diagnosis of diabetic foot. The detection is based on various criteria. Some of them compare the temperature between the studied feet and the healthy participant’s feet, while others compare the relative difference of the temperature in the homologous ROI points of the two feet. Also, there are a few studies that use unsupervised techniques to cluster the severity risk of diabetic foot ulcers for a single foot (Khandakar et al. (2022)). Table 6 provides a summary on the thermal/infrared imaging systems used for diabetic foot applications, along with the analysis procedure followed and the findings of each study.

**TABLE 6:** Summary of the literature for diabetic foot monitoring using imagery data from thermal sensors.

Study	Basic Equipment and Environmental Conditions	Participants (number and characteristics) or dataset description	Algorithms and methods	Experimental Results and performance evaluation
van Netten etal.,(2013)	RGB camera, Canon Eos 40D with *EF* ^−*s*^17 − −85*mm* lens, and IR Thermal camera, FLIR SC305	15 diabetes patients	Δ*T* between feet for each ROI (Kruskal–Wallis test).	Δ*T* < 2, small temperature differences. Δ*T* > 2°*C*, a noninfected and nonischemic foot ulcer. Δ*T* > 3°*C*, a foot ulcer with osteomyelitis or a Charcot foot.
Liu etal.,(2015)	RGB camera, Canon EOS 40D and IR camera, FLIR SC305	76 diabetes patients (Type I Diabetis Mellitus [7 patients] and Type II [69 patients])	K-means clustering, EM algorithm for foot segmentation, registration, and detection	Δ*T* between contralateral points, Average, SD. Segmentation technique in the thermal images achieve accuracy 97.9% ± 1.1% and 98.3% ± 0.5%.
Petrova etal.,(2018)	thermal imaging device, (Photometrix Imaging Ltd) and infrared spot thermometer	105 participants (52 males and 53 females; age range 18 to 69 years)	Threshold for image segmentation. Repeated measurements Regression analysis	Δ*T* for each predefined ROI 1^*st*^ toe 0.04, 4^*th*^ toe 0.03, 1^*st*^ metatarsal − 0.01, 3^*rd*^ metatarsal 0.11 and 5^*th*^ metatarsal 0.21
Maldonado etal.,(2020)	IR FLUKE TI32 IRT camera	17 volunteers (108 images)	Mask R–CNN model for segmentation	Temperature differences detection and classification as ulcerous if >2.2°C and necrotic if <−2.2°C .
Eid etal.,(2018)	IR FLIR ONE thermal camera, Additional Equipment: Samsung Note five smartphone, temperature and humidity sensor, etc.	50 volunteers (without any complications, with local foot complications, with deep ulcer or Charcot’s foot, with amputation)	Classifiers used for segmentation: (i) k-Nearest Neighbor; (ii) Support vector machine, and Decision tree	Mean absolute Δ*T* between the corresponding points of both feet divided in classes: Δ*T* < 1.5°*C*, 1.5°*C* < Δ*T* > 2°*C*, 2°*C* < Δ*T* > 3°*C*, Δ*T* > 3°*C*.
Cruz-Vega etal.,(2020)	-	110 thermograms of DM subjects	Automatic segmentation. CNNs models for multi-class classification of the thermograms. Five categories of the temperature change in foot	Five categories (medial plantar artery, lateral plantar artery, medial calcaneal artery, and lateral calcaneal artery) of the relative change in temperature of the plantar regions.
Vardasca etal., (2019b)	FLIR thermal camera (FPA sensor array(320x240), NETD of <50mK at 30°*C*)	39 patients:(14 appear ischaemic wound, 25 had a healing wound)	k-NN, SVM, ANN techniques	k-NN with accuracy 0.81, 0.80 specificity and 1.0 sensitivity
Adam etal.,(2018)	Thermographic System VarioCAM© hr head 680/30mm positioned at 1m distance from the feet	51 healthy individuals and 66 patients with diabetes (the half of them having neuropathy)	Thermograms image segmentation with wavelet transform. Feature extraction. Image classification using k-NN	The thermal image analysis method succeeds 93.16% accuracy, 90.91% sensitivity and 98.04% specificity
Petrova etal.,(2020)	standard digital RGB + Portable Infrared thermal camera	110 people with diabetes. 61 for the control group, 49 for the intervention group n = 49, study period 12 months	Uni– and multivariate modelling of the likelihood of ulcer recurrence during the studied period	Change in temperature between ≥2.2°C .
van Doremalen etal.,(2020)	3 thermal infrared cameras from smartphone devices. medical 3D imaging system	8 patients with diabetic foot ulcer	Creation of 3D thermographs using a passive photogrammetry technique	3D modelling from the thermal foot images to assess the temperature of the diabetic foot.
Astasio-Picado etal.,(2018)	Infrared camera model FLIR E60bx	277 patients (138 men-139 women), in four groups (with neuropathy, vasculopathy, neurovasculopathy, and healthy feet)	IBM SPSS Statistics statistical program	Lower T values are observed under the ROIs in both feet of the patients in the neuropathy, vasculopathy, and neurovasculopathy groups relative to the healthy feet group.
Yavuz etal.,(2019)	Infrared thermal cameras FLIR T650sc (FLIR Systems Inc, Wilsonville, Oregon) or Fluke TiR2 (Fluke Corp, Everett, Washington)	37 participants: 9 with DFU, 14 with diabetic neuropathy (DN), and 14 with nonneuropathic control (DC).	Mean T was determined in four regions-hallux and medial, central, and lateral forefoot- linear models with specified contrasts among the DFU, DN, and DC groups.	Mean T in each foot region was higher than 30.0°*C* in DFU and DN and lower than 30.0°*C* in the DC group. Mean differences werehigher in the DFU and DC groups, ranging from 3.2°*C* in the medial forefoot to 4.9°*C* in the hallux.
Fraiwan etal.,(2018)	mobile thermal camera. homogeneous background, room temperature (20 − −25°*C*)	-	Image pre-processing. Ulcers detection. Foot sole segmentation method Thresholding (Otsu) (temperature matrix).	Test whether Δ*T* is greater than 2.2°*C*.
Neves etal.,(2015)	High-resolution infrared camera (FLIR Systems Inc. Model SC 2000; 320 × 240 pixels)	44 volunteers in total (22 women and 22 men; 66.70 ± 6.26 years of age) with type 2 diabetes (diagnosed at 11.84 ± 8.22 years)	ROIs: first finger, fifth finger and the heel. Pearson’s correlations between the variables (body mass, body height, BMI and body fat) and Δ*T* values per ROI.	ROIs higher Δ*T* ≥ 2.20°*C*. A positive association is observed either of BMI (*r* = 0.399, *p* = 0.007) either of body fat percentage (*r* = 0.432, *p* = 0.003), with diabetic foot risk in patients with type 2 diabetes.
Keenan etal.,(2017)	FLIR A325 infrared camera. Spatial resolution (instantaneous field of view) of 1.36 *mrad* and sensitivity of 70 *mK* at 30°*C*.	11 patients with non-infected DFUs and 3 patients with non-diabetic wounds.	Measurements of the different temperatures over the ROI. Emissivity metric was calculated per pixel.	The emissivity of wounds has range 0.01 − 0.03 with an average value of 0.9 ± 0.03, and with lower values at wound edges (on average 0.02 lower than intact skin).
Ilo etal.,(2020)	IR camera (FLIR A325sc) with 320 × 240 pixels and 0.05°*C* thermal resolution	118 patients with DM and 93 healthy	-	-.
Munadi etal.,(2022)	two infrared cameras (FLIR E60 and FLIR E6)	167 plantar thermograms (122 diabetic and 45 non-diabetic subjects)	fused CNNs for classification of diabetic or healthy images	accuracy, recall, precision, F-measure metrics

## 6 Discussion

In this paper we focused on comparing noninvasive techniques and approaches for DFU monitoring, and to highlight their advantages and disadvantages. There are multiple sensors, which can support efficiently the monitoring process, resulting in an improvement of patient’s life. Yet, the type of raw data provided, including spectral range and resolution, remains a topic of debate, susceptible to limitations related to cost and portability of the monitoring devices. In total, four crucial monitoring factors have been identified namely: a) glucose levels, b) foot deformity/wounds, c) temperature, and d) hemoglobin concentration. All these factors can be currently monitored with the usage of various sensors. Currently, none of the existing sensors that are commercially available supports a holistic self monitoring, including measurements for all the above mentioned factors. In particularly, RGB sensors can be used to distinguish deformities on the skin, NIR spectroscopy (700*nm* − 1300 *nm*) can capture haemoglobin saturation, thermal infrared spectroscopy can detect hyperthermia (or hypothermia), among ROIs of a foot, and the Mid-IR absorbance (5.7 *μm* − 9.3 *μm*) spectrum provides rich information about the proteomics, lipidomics, and metabolomics (e.g., glucose).

### 6.1 Challenges on data acquisition

As regards the cost-effectiveness of the sensory systems, it heavily depends on the type of sensors that each system is equipped with. On the one hand, self-monitoring devices are usually equipped with RGB cameras or with sensors in thermal infrared spectrum González-Pérez et al. (2021) and both cases are considered low-cost. On the other hand, HSI equipment is more expensive than the traditional methods and it is not recommended for self-monitoring cases López-Moral et al. (2022).

High quality visible and infrared (thermography) images can be acquired through portable devices (e.g., cell-phones or even lightweight and low-cost equipment). As regards hyperspectral imagery data in DFU monitoring, the high volume of HSI images is an obstacle for applying these techniques to be used in conjunction with the smartphone applications. However, recently there are appeared relevant studies that propose solutions where acquiring HSI images through smart phone devices could be feasible Stuart et al. (2021). Hyperspectral images are capable of accurate spectral and spatial data collection and have the potential for future deployment combined with smartphones for DFU monitoring applications. Portability and user-friendly setup of this devices is of major concern, in order to make it a valuable instrument to assist the decision process of the doctors. However, these solutions are yet immature and future development and improvements in this area of research is necessary in order to provide commercial products and ready-to-use solutions.

### 6.2 Challenges on machine learning algorithms

Machine learning and computer vision methods, including detection, segmentation and classification, are used to analyse the diabetic foot ulcer images. Usually, DFU is addressed as a binary classification problem; we either have a DFU or not, or we have the appearance of a specific factor, correlated to DFU. To assess the usability of these models, it is important to incorporate them on mobile and cloud or even edge-based technologies. Given the significant and growing impact of DFU, mobile health solutions that target this condition could assist in improving patient quality of life (Cassidy et al., 2022). Taking this into consideration, trustworthiness in AI solutions for DFU monitoring is an important aspect.

The performance evaluation of the ML model aims to assess the method’s effectiveness in the accurate detection of DFU. There are various metrics for ML models, which can be often combined to evaluate a model (Zhang et al., 2022). Cross-validation technique is also an important part of the evaluation process, because it provides an insight into the model’s precision level and is a necessary part of the algorithmic process to ensure the models’ stability and to define the confidence intervals of the proposed method. The machine learning approaches should always keep the trade-off between the complexity of the model/architecture and the accuracy improvement. CNNs have had a great success in the recent past, due to the advent of faster GPUs and vast amount of memory access, however, deploying deep learning applications on the edge has constrains as regards the computational resources. Thus, it is important to make models feasible for constrained devices, such as mobile phones. In health care systems, deep-learning models largely rely on sufficient and diverse training data gathered from patients. However, leveraging AI-based technologies to improve the management of diabetic foot ulcers is usually challenging due to limits arising from the legislation on patient’s privacy and data security. Thus, safety and privacy issues may imply fewer data available; therefore, additional challenges for training supervised AI algorithms with good performance appear. However, recently, federated schemes have emerged as the state-of-the-art techniques in order to achieve personalized recommendations in health care systems with state-of-the-art accuracy, while ensuring privacy preservation for the patient (Rieke et al., 2020).

## Appendix A

Abbreviations

ANN, Artificial neural network; BMI; Body mass index; CCD, Charge-coupled device; CNN, Convolutional neural network; DFU, Diabetic foot ulcer; DM, Diabetes Mellitus; EC-QCL, External cavity-quantum cascade laser; EM, Expectation maximization; GAN, Generative Adverserial neural network; HSI, hyperspectral imaging; IRT; Infrared thermography, k-NN; k-nearest neighbours; LCTF, Liquid crystal tunable filter; MaP, Mean average precision; MIR, Mid - infrared; NIR, Near - infrared; PAD, Peripheral arterial disease; PCA, Principal component analysis; PSNR, Peak signal-to-noise-ratio; QCL, Quantum cascade lasers; RCNN, Region-based Convolutional Neural Network; RMSE, Root Mean Square Error; ROI, Region of interest; SSIM, Structural similarity index measure; SVM, Support vector machines; VSI, Visual spectrum imaging.

## References

[B1] Adam M. Ng E. Y. Oh S. L. Heng M. L. Hagiwara Y. Tan J. H. (2018). Automated detection of diabetic foot with and without neuropathy using double density-dual tree-complex wavelet transform on foot thermograms. Infrared Phys. Technol. 92, 270–279. 10.1016/j.infrared.2018.06.010

[B2] Aitchison R. T. Ward L. Kennedy G. J. Shu X. Mansfield D. C. Shahani U. (2018). Measuring visual cortical oxygenation in diabetes using functional near-infrared spectroscopy. Acta Diabetol. 55, 1181–1189. 10.1007/s00592-018-1200-5 30083981 PMC6182359

[B3] Alatrany A. S. Hussain A. Alatrany S. S. Al-Jumaily D. (2022). “Application of deep learning autoencoders as features extractor of diabetic foot ulcer images,” in International conference on intelligent computing (Germany: Springer), 129–140.

[B4] Alzubaidi L. Al-Amidie M. Al-Asadi A. Humaidi A. J. Al-Shamma O. Fadhel M. A. (2021). Novel transfer learning approach for medical imaging with limited labeled data. Cancers 13, 1590. 10.3390/cancers13071590 33808207 PMC8036379

[B5] Armstrong D. G. Boulton A. J. Bus S. A. (2017). Diabetic foot ulcers and their recurrence. N. Engl. J. Med. 376, 2367–2375. 10.1056/NEJMra1615439 28614678

[B6] Astasio-Picado A. Martínez E. E. Nova A. M. Rodríguez R. S. Gómez-Martín B. (2018). Thermal map of the diabetic foot using infrared thermography. Infrared Phys. Technol. 93, 59–62. 10.1016/j.infrared.2018.07.008

[B7] Bus S. A. van Netten J. J. (2016). A shift in priority in diabetic foot care and research: 75% of foot ulcers are preventable. Diabetes. Metab. Res. Rev. 32, 195–200. 10.1002/dmrr.2738 26452160

[B8] Cai Z. Vasconcelos N. (2019). Cascade r-cnn: High quality object detection and instance segmentation. IEEE Trans. Pattern Anal. Mach. Intell. 43, 1483–1498. 10.1109/TPAMI.2019.2956516 31794388

[B9] Cassidy B. Reeves N. D. Pappachan J. M. Ahmad N. Haycocks S. Gillespie D. (2022). A cloud-based deep learning framework for remote detection of diabetic foot ulcers. IEEE Pervasive Comput. 21, 78–86. 10.1109/mprv.2021.3135686

[B10] Cassidy B. Reeves N. D. Pappachan J. M. Gillespie D. O’Shea C. Rajbhandari S. (2021). The dfuc 2020 dataset: Analysis towards diabetic foot ulcer detection. touchREV. Endocrinol. 17, 5–11. 10.17925/EE.2021.17.1.5 35118441 PMC8320006

[B11] Cheng Y. Zu P. Zhao J. Shi L. Shi H. Zhang M. (2021). Differences in initial versus recurrent diabetic foot ulcers at a specialized tertiary diabetic foot care center in China. J. Int. Med. Res. 49, 030006052098739. 10.1177/0300060520987398 PMC7829526 33472497

[B12] Ciurczak E. W. Igne B. (2014). Pharmaceutical and medical applications of near-infrared spectroscopy. Florida: CRC Press.

[B13] Cousart T. H. Handley M. (2017). Implementing diabetic foot care in the primary care setting. J. Nurse Pract. 13, e129–e132. 10.1016/j.nurpra.2016.11.009

[B14] Cruz-Vega I. Hernandez-Contreras D. Peregrina-Barreto H. Rangel-Magdaleno J. d. J. Ramirez-Cortes J. M. (2020). Deep learning classification for diabetic foot thermograms. Sensors 20, 1762. 10.3390/s20061762 PMC7147707 32235780

[B15] Cui C. Thurnhofer-Hemsi K. Soroushmehr R. Mishra A. Gryak J. Domínguez E. (2019). Diabetic wound segmentation using convolutional neural networks. In 2019 41st Annual International Conference of the IEEE Engineering in Medicine and Biology Society (EMBC) (IEEE), 23-27 July 2019, Berlin, Germany, 1002–1005. 10.1109/EMBC.2019.8856665 PMC8446935 31946062

[B16] Das S. K. Roy P. Mishra A. K. (2022). Fusion of handcrafted and deep convolutional neural network features for effective identification of diabetic foot ulcer. Concurrency Comput. 34, e6690. 10.1002/cpe.6690

[B17] Davradou A. Protopapadakis E. Kaselimi M. Doulamis A. Doulamis N. (2022). “Diabetic foot ulcers monitoring by employing super resolution and noise reduction deep learning techniques,” in Proceedings of the 15th International Conference on PErvasive Technologies Related to Assistive Environments (New York, NY: Association for Computing Machinery), 83–88. 10.1145/3529190.3529214

[B18] Eid M. M. Yousef R. N. Mohamed M. A. (2018). A proposed automated system to classify diabetic foot from thermography. Int. J. Sci. Eng. Res. 9, 371–381.

[B19] Fei B. (2020). Hyperspectral imaging in medical applications. Data Handl. Sci. Technol. 32, 523–565. Elsevier. 10.1016/B978-0-444-63977-6.00021-3

[B20] Fraiwan L. Ninan J. Al-Khodari M. (2018). Mobile application for ulcer detection. Open Biomed. Eng. J. 12, 16–26. 10.2174/1874120701812010016 30069252 PMC6048827

[B21] González-Pérez S. Perea Ström D. Arteaga-Marrero N. Luque C. Sidrach-Cardona I. Villa E. (2021). Assessment of registration methods for thermal infrared and visible images for diabetic foot monitoring. Sensors 21, 2264. 10.3390/s21072264 33804926 PMC8037427

[B22] Goyal M. Reeves N. D. Davison A. K. Rajbhandari S. Spragg J. Yap M. H. (2018). Dfunet: Convolutional neural networks for diabetic foot ulcer classification. IEEE Trans. Emerg. Top. Comput. Intell. 4, 728–739. 10.1109/tetci.2018.2866254

[B23] Goyal M. Reeves N. D. Rajbhandari S. Ahmad N. Wang C. Yap M. H. (2020). Recognition of ischaemia and infection in diabetic foot ulcers: Dataset and techniques. Comput. Biol. Med. 117, 103616. 10.1016/j.compbiomed.2020.103616 32072964

[B24] Greenman R. L. Panasyuk S. Wang X. Lyons T. E. Dinh T. Longoria L. (2005). Early changes in the skin microcirculation and muscle metabolism of the diabetic foot. Lancet 366, 1711–1717. 10.1016/S0140-6736(05)67696-9 16291064

[B25] Gurjarpadhye A. A. Parekh M. B. Dubnika A. Rajadas J. Inayathullah M. (2015). Infrared imaging tools for diagnostic applications in dermatology. SM J. Clin. Med. Imaging 1, 1–5. 26691203 PMC4683617

[B26] Hillen B. Pfirrmann D. Nägele M. Simon P. (2020). Infrared thermography in exercise physiology: The dawning of exercise radiomics. Sports Med. 50, 263–282. 10.1007/s40279-019-01210-w 31734882

[B27] Ilo A. Romsi P. Mäkelä J. (2020). Infrared thermography and vascular disorders in diabetic feet. J. Diabetes Sci. Technol. 14, 28–36. 10.1177/1932296819871270 31452395 PMC7189167

[B28] Jaly I. Iyengar K. Bahl S. Hughes T. Vaishya R. (2020). Redefining diabetic foot disease management service during Covid-19 pandemic. Diabetes Metab. Syndr. 14, 833–838. 10.1016/j.dsx.2020.06.023 32540738 PMC7289094

[B29] Jeffcoate W. Clark D. Savic N. Rodmell P. Hinchliffe R. Musgrove A. (2015). Use of hsi to measure oxygen saturation in the lower limb and its correlation with healing of foot ulcers in diabetes. Diabet. Med. 32, 798–802. 10.1111/dme.12778 25864911

[B30] Ji X. Akiyarna Y. Yamada Y. Okamoto S. Hayashi H. (2020). Development of deep clustering model to stratify occurrence risk of diabetic foot ulcers based on foot pressure patterns and clinical indices. In 2020 IEEE International Joint Conference on Biometrics (IJCB) (IEEE), 01 October 2020, Houston, TX, USA, 1–8

[B31] Kateel R. Augustine A. J. Prabhu S. Ullal S. Pai M. Adhikari P. (2018). Clinical and microbiological profile of diabetic foot ulcer patients in a tertiary care hospital. Diabetes Metab. Syndr. 12, 27–30. 10.1016/j.dsx.2017.08.008 28867530

[B32] Keenan E. Gethin G. Flynn L. Watterson D. O’Connor G. M. (2017). Enhanced thermal imaging of wound tissue for better clinical decision making. Physiol. Meas. 38, 1104–1115. 10.1088/1361-6579/aa6ea0 28430667

[B33] Kendrick C. Cassidy B. Pappachan J. M. O’Shea C. Fernandez C. J. Chacko E. (2022). Translating clinical delineation of diabetic foot ulcers into machine interpretable segmentation. arXiv preprint arXiv:2204.11618

[B34] Khagi B. Lee C. G. Kwon G.-R. (2018). Alzheimer’s disease classification from brain mri based on transfer learning from cnn. In 2018 11th biomedical engineering international conference (BMEiCON) (IEEE), 21-24 November 2018, Thailand,

[B35] Khan M. J. Khan H. S. Yousaf A. Khurshid K. Abbas A. (2018). Modern trends in hyperspectral image analysis: A review. Ieee Access 6, 14118–14129. 10.1109/access.2018.2812999

[B36] Khandakar A. Chowdhury M. E. Reaz M. B. I. Ali S. H. M. Kiranyaz S. Rahman T. (2022). A novel machine learning approach for severity classification of diabetic foot complications using thermogram images. Sensors 22, 4249. 10.3390/s22114249 35684870 PMC9185274

[B37] Khaodhiar L. Dinh T. Schomacker K. T. Panasyuk S. V. Freeman J. E. Lew R. (2007). The use of medical hyperspectral technology to evaluate microcirculatory changes in diabetic foot ulcers and to predict clinical outcomes. Diabetes care 30, 903–910. 10.2337/dc06-2209 17303790

[B38] Kottmann J. Rey J. M. Luginbühl J. Reichmann E. Sigrist M. W. (2012). Glucose sensing in human epidermis using mid-infrared photoacoustic detection. Biomed. Opt. Express 3, 667–680. 10.1364/BOE.3.000667 22574256 PMC3345797

[B39] Lim J. Z. M. Ng N. S. L. Thomas C. (2017). Prevention and treatment of diabetic foot ulcers. J. R. Soc. Med. 110, 104–109. 10.1177/0141076816688346 28116957 PMC5349377

[B40] Liu C. van Netten J. J. Van Baal J. G. Bus S. A. van Der Heijden F. (2015). Automatic detection of diabetic foot complications with infrared thermography by asymmetric analysis. J. Biomed. Opt. 20, 026003. 10.1117/1.JBO.20.2.026003 25671671

[B41] López-Moral M. García-Álvarez Y. Molines-Barroso R. J. Tardáguila-García A. García-Madrid M. Lázaro-Martínez J. L. (2022). A comparison of hyperspectral imaging with routine vascular noninvasive techniques to assess the healing prognosis in patients with diabetic foot ulcers. J. Vasc. Surg. 75, 255–261. 10.1016/j.jvs.2021.07.123 34314832

[B42] Makantasis K. Georgogiannis A. Voulodimos A. Georgoulas I. Doulamis A. Doulamis N. (2021). Rank-r fnn: A tensor-based learning model for high-order data classification. IEEE Access 9, 58609–58620. 10.1109/access.2021.3072973

[B43] Maldonado H. Bayareh R. Torres I. Vera A. Gutiérrez J. Leija L. (2020). Automatic detection of risk zones in diabetic foot soles by processing thermographic images taken in an uncontrolled environment. Infrared Phys. Technol. 105, 103187. 10.1016/j.infrared.2020.103187

[B44] Mejaiti N. van Netten J. J. Dijkgraaf M. G. van Baal J. G. Busch-Westbroek T. E. Bus S. A. (2018). The cost-effectiveness and cost-utility of at-home infrared temperature monitoring in reducing the incidence of foot ulcer recurrence in patients with diabetes (diatemp): Study protocol for a randomized controlled trial. Trials 19, 1–12. 10.1186/s13063-018-2890-2 30249296 PMC6154404

[B45] Munadi K. Saddami K. Oktiana M. Roslidar R. Muchtar K. Melinda M. (2022). A deep learning method for early detection of diabetic foot using decision fusion and thermal images. Appl. Sci. 12, 7524. 10.3390/app12157524

[B46] Najafi B. Mishra R. (2021). Harnessing digital health technologies to remotely manage diabetic foot syndrome: A narrative review. Medicina 57, 377. 10.3390/medicina57040377 33919683 PMC8069817

[B47] Najafi B. Reeves N. D. Armstrong D. G. (2020). Leveraging smart technologies to improve the management of diabetic foot ulcers and extend ulcer-free days in remission. Diabetes. Metab. Res. Rev. 36, e3239. 10.1002/dmrr.3239 31909547

[B48] Nanda R. Nath A. Patel S. Mohapatra E. (2022). Machine learning algorithm to evaluate risk factors of diabetic foot ulcers and its severity. Med. Biol. Eng. Comput. 60, 2349–2357. 10.1007/s11517-022-02617-w 35751828

[B49] Neves E. B. Almeida A. J. Rosa C. Vilaça-Alves J. Reis V. M. Mendes R. (2015). Anthropometric profile and diabetic foot risk: A cross-sectional study using thermography. 2015 37th Annu. Int. Conf. IEEE Eng. Med. Biol. Soc. (EMBC) (IEEE) 2015, 1–3. 10.1109/EMBC.2015.7445519 28556779

[B50] Nouvong A. Hoogwerf B. Mohler E. Davis B. Tajaddini A. Medenilla E. (2009). Evaluation of diabetic foot ulcer healing with hyperspectral imaging of oxyhemoglobin and deoxyhemoglobin. Diabetes care 32, 2056–2061. 10.2337/dc08-2246 19641161 PMC2768226

[B51] Ohura N. Mitsuno R. Sakisaka M. Terabe Y. Morishige Y. Uchiyama A. (2019). Convolutional neural networks for wound detection: The role of artificial intelligence in wound care. J. Wound Care 28, S13–S24. 10.12968/jowc.2019.28.Sup10.S13 31600101

[B52] Pasquini C. (2003). Near infrared spectroscopy: Fundamentals, practical aspects and analytical applications. J. Braz. Chem. Soc. 14, 198–219. 10.1590/s0103-50532003000200006

[B53] Petrova N. Donaldson N. Tang W. MacDonald A. Allen J. Lomas C. (2020). Infrared thermography and ulcer prevention in the high-risk diabetic foot: Data from a single-blind multicentre controlled clinical trial. Diabet. Med. 37, 95–104. 10.1111/dme.14152 31629373

[B54] Petrova N. Whittam A. MacDonald A. Ainarkar S. Donaldson A. Bevans J. (2018). Reliability of a novel thermal imaging system for temperature assessment of healthy feet. J. Foot Ankle Res. 11, 22–26. 10.1186/s13047-018-0266-1 29854007 PMC5975531

[B55] Protopapadakis E. Doulamis A. Doulamis N. Maltezos E. (2021). Stacked autoencoders driven by semi-supervised learning for building extraction from near infrared remote sensing imagery. Remote Sens. 13, 371. 10.3390/rs13030371

[B56] Rania N. Douzi H. Yves L. Sylvie T. (2020). “Semantic segmentation of diabetic foot ulcer images: Dealing with small dataset in dl approaches,” in International conference on image and signal processing (Germany: Springer), 162–169.

[B57] Rieke N. Hancox J. Li W. Milletari F. Roth H. R. Albarqouni S. (2020). The future of digital health with federated learning. NPJ Digit. Med. 3, 1–7. 10.1038/s41746-020-00323-1 33015372 PMC7490367

[B58] Rubins U. Marcinkevics Z. Cimurs J. Saknite I. Kviesis-Kipge E. Grabovskis A. (2019). Multimodal device for real-time monitoring of skin oxygen saturation and microcirculation function. Biosensors 9, 97. 10.3390/bios9030097 PMC6784356 31382463

[B59] Saito H. Ishikawa T. Tanabe J. Kobayashi S. Moroi J. (2018). Bedside assessment of regional cerebral perfusion using near-infrared spectroscopy and indocyanine green in patients with atherosclerotic occlusive disease. Sci. Rep. 8, 1242–1248. 10.1038/s41598-018-19668-5 29352217 PMC5775286

[B60] Salman I. N. Wadood S. A. Abualkasem B. A. (2017). Low hemoglobin levels in infected diabetic foot ulcer. Age (y) 36, 40–65.

[B61] Sarawade A. A. Charniya N. N. (2018). Infrared thermography and its applications: A review. In 2018 3rd International Conference on Communication and Electronics Systems (ICCES) (IEEE), 15-16 October 2018, Coimbatore, 280–285.

[B62] Schaper N. Van Netten J. Apelqvist J. Lipsky B. Bakker K. Diabetic I. (2016). Prevention and management of foot problems in diabetes: A summary guidance for daily practice 2015, based on the iwgdf guidance documents. Diabetes. Metab. Res. Rev. 32, 7–15. 10.1002/dmrr.2695 26335366

[B63] Stuart M. B. McGonigle A. J. Davies M. Hobbs M. J. Boone N. A. Stanger L. R. (2021). Low-cost hyperspectral imaging with a smartphone. J. Imaging 7, 136. 10.3390/jimaging7080136 34460772 PMC8404918

[B64] Tulloch J. Zamani R. Akrami M. (2020). Machine learning in the prevention, diagnosis and management of diabetic foot ulcers: A systematic review. IEEE Access 8, 198977–199000. 10.1109/access.2020.3035327

[B65] Tzortzis I. N. Davradou A. Protopapadakis E. Kaselimi M. Doulamis N. Angeli A. (2022). Unsupervised diabetic foot monitoring techniques. In Proceedings of the 15th International Conference on PErvasive Technologies Related to Assistive Environments, March 10, 2022, Kerkira. 608–614.

[B66] van Doremalen R. F. van Netten J. J. van Baal J. G. Vollenbroek M. M. Heijden F. (2020). Infrared 3d thermography for inflammation detection in diabetic foot disease: A proof of concept. J. Diabetes Sci. Technol. 14, 46–54. 10.1177/1932296819854062 31200612 PMC7189170

[B67] van Netten J. J. Clark D. Lazzarini P. A. Janda M. Reed L. F. (2017). The validity and reliability of remote diabetic foot ulcer assessment using mobile phone images. Sci. Rep. 7, 1–10. 10.1038/s41598-017-09828-4 28842686 PMC5573347

[B68] [Dataset] van Netten J. J. van Baal J. G. Liu C. van Der Heijden F. Bus S. A. (2013). Infrared thermal imaging for automated detection of diabetic foot complications. J. Diabetes Sci. Technol. 7, 1122–1129. 10.1177/193229681300700504 24124937 PMC3876354

[B69] Vardasca R. Magalhães C. Seixas A. Carvalho R. Mendes J. (2019b). Diabetic foot monitoring using dynamic thermography and ai classifiers. In Third Quantitative Infrared Thermography Asian Conference, July 2019: Tokyo, Japan

[B70] Vardasca R. Magalhaes C. Silva P. Abreu P. Mendes J. Restivo M. T. (2019a). Biomedical musculoskeletal applications of infrared thermal imaging on arm and forearm: A systematic review. J. Therm. Biol. 82, 164–177. 10.1016/j.jtherbio.2019.04.008 31128644

[B71] Voulodimos A. Doulamis N. Doulamis A. Protopapadakis E. (2018). Deep learning for computer vision: A brief review. Comput. Intell. Neurosci. 2018. 10.1155/2018/7068349 PMC5816885 29487619

[B72] Wang L. Pedersen P. C. Agu E. Strong D. M. Tulu B. (2019). Boundary determination of foot ulcer images by applying the associative hierarchical random field framework. J. Med. Imaging 6, 024002. 10.1117/1.JMI.6.2.024002 PMC6475526 31037245

[B73] Xie P. Li Y. Deng B. Du C. Rui S. Deng W. (2021). An explainable machine learning model for predicting in-hospital amputation rate of patients with diabetic foot ulcer. Int. Wound J. 19, 910–918. 10.1111/iwj.13691 34520110 PMC9013600

[B74] Xu Y. Han K. Zhou Y. Wu J. Xie X. Xiang W. (2021). Classification of diabetic foot ulcers using class knowledge banks. Front. Bioeng. Biotechnol. 9, 811028. 10.3389/fbioe.2021.811028 35295708 PMC8918844

[B75] Yang Q. Sun S. Jeffcoate W. J. Clark D. J. Musgove A. Game F. L. (2018). Investigation of the performance of hyperspectral imaging by principal component analysis in the prediction of healing of diabetic foot ulcers. J. Imaging 4, 144. 10.3390/jimaging4120144

[B76] Yap M. H. Cassidy B. Kendrick C. (2022). Diabetic foot ulcers grand challenge. Germany: Springer.

[B77] Yap M. H. Chatwin K. E. Ng C.-C. Abbott C. A. Bowling F. L. Rajbhandari S. (2018). A new mobile application for standardizing diabetic foot images. J. Diabetes Sci. Technol. 12, 169–173. 10.1177/1932296817713761 28637356 PMC5761973

[B78] Yap M. H. Hachiuma R. Alavi A. Brüngel R. Cassidy B. Goyal M. (2021). Deep learning in diabetic foot ulcers detection: A comprehensive evaluation. Comput. Biol. Med. 135, 104596. 10.1016/j.compbiomed.2021.104596 34247133

[B79] Yavuz M. Ersen A. Hartos J. Lavery L. A. Wukich D. K. Hirschman G. B. (2019). Temperature as a causative factor in diabetic foot ulcers: A call to revisit ulceration pathomechanics. J. Am. Podiatr. Med. Assoc. 109, 345–350. 10.7547/17-131 30427732

[B80] Yazdanpanah L. Shahbazian H. Nazari I. Arti H. R. Ahmadi F. Mohammadianinejad S. E. (2018). Incidence and risk factors of diabetic foot ulcer: A population-based diabetic foot cohort (adfc study)—two-year follow-up study. Int. J. Endocrinol. 2018, 7631659. 10.1155/2018/7631659 29736169 PMC5875034

[B81] Yudovsky D. Nouvong A. Pilon L. (2010). Hyperspectral imaging in diabetic foot wound care. J. Diabetes Sci. Technol. 4, 1099–1113. 10.1177/193229681000400508 20920429 PMC2956800

[B82] Zhang J. Qiu Y. Peng L. Zhou Q. Wang Z. Qi M. (2022). A comprehensive review of methods based on deep learning for diabetes-related foot ulcers. Front. Endocrinol. 13, 945020. 10.3389/fendo.2022.945020 PMC9394750 36004341

[B83] Zhang Q. Yuan Q. Li J. Sun F. Zhang L. (2020). Deep spatio-spectral bayesian posterior for hyperspectral image non-iid noise removal. ISPRS J. Photogrammetry Remote Sens. 164, 125–137. 10.1016/j.isprsjprs.2020.04.010

